# Commentary: Pediatric Surgery in Low‐Income Countries—From Neglect to Necessity

**DOI:** 10.1002/wjs.70113

**Published:** 2025-09-28

**Authors:** Zipporah Gathuya, Elizabeth T. Drum

**Affiliations:** ^1^ The Nairobi Hospital Nairobi Kenya; ^2^ Children's Hospital of Philadelphia Perelman School of Medicine University of Pennsylvania Philadelphia Pennsylvania USA

In this issue [1], Hsu et al. present a landmark multicenter study evaluating pediatric surgical outcomes across 17 hospitals in 11 African countries between 2018 and 2023. This study is a significant contribution to global surgery literature—both for its scale and its relevance. For low‐income countries (LICs), where pediatric surgery remains persistently underprioritized, underfunded, and under‐researched, these findings help to fill a critical data void.

Importantly, this is not just a study about numbers; it is a study about systems. Hsu et al. move beyond descriptive epidemiology to offer a compelling narrative about the structural limitations that drive poor outcomes. As is often the case in global health, the knowledge and technical capacity to manage surgical disease in children exists. What remains lacking is the systemic will—and the coordinated investment—to translate this knowledge into accessible, timely, and safe care.

Surgery does not occur in a vacuum. Its effectiveness is tightly bound to the availability of supportive services: anesthesia, nursing, radiology, pharmacy, and essential equipment. Partners in Health famously refers to the “Five S’s”—*staff, stuff, space, systems, and social* *support* [2]—and this study illustrates how fragility in any one of these domains can lead to avoidable childhood mortality.

Perhaps the most sobering finding is the high neonatal mortality rate—16.8% in this cohort—compared to 0.3%–2.4% in high‐income countries (HICs). Much of this mortality was due to congenital anomalies, many of which are survivable if treated appropriately. These elevated death rates reflect delayed presentation, inadequate referral systems, limited neonatal intensive care, and a severe shortage of pediatric surgical and anesthetic expertise. Even basic perioperative tools—such as neonatal ventilators or safe anesthetic agents—are often unreliable or altogether absent in many LIC facilities.

It is also notable that these outcomes were observed in hospitals equipped by Kids Operating Room (KidsOR), a non‐governmental organization (NGO) providing infrastructure and training to improve pediatric surgical care. If outcomes are this stark in better‐resourced environments, one can only imagine the situation in under‐resourced or non‐dedicated surgical facilities. Furthermore, as the authors note, KidsOR facilities focus primarily on general pediatric surgery—highlighting a pressing need to expand care across neurosurgery, orthopedics, and other subspecialties.

Another striking finding is the disproportionate mortality from emergency surgeries. Although emergency procedures made up only 37% of the total, they accounted for 88% of all deaths, with a 13‐fold higher mortality rate than elective operations. This reflects systemic gaps in emergency preparedness, delayed recognition and referral, transportation barriers, and a lack of intensive care for acutely ill children.

This data reinforces the relevance of the “three delays” framework [3] in pediatric surgical care:

1. Delay in seeking care
2. Delay in reaching care
3. Delay in receiving timely and appropriate treatment

Each delay compounds the risk of adverse outcomes, and each requires intervention at multiple levels—from community engagement to national policy.

A critical workforce imbalance also emerges: the stark underrepresentation of pediatric anesthesiologists. While many LICs have made strides in training pediatric surgeons, anesthesia remains a bottleneck. Addressing this gap will require investment in specialist training programs, task‐sharing strategies, and the development of context‐appropriate short courses. Pediatric anesthesiologists are not just essential providers; they are leaders in ensuring perioperative safety.

Although the contribution of NGOs such as KidsOR is commendable, this study prompts deeper reflection. What are the outcomes in hospitals without such support? Do NGOs shift government attention or funding away from system‐wide investment—or can they serve as catalysts for broader change? How can we ensure that standards of care established in NGO‐supported facilities are equitably extended to all children, regardless of geography or socioeconomic status?

The urgency of these questions is magnified by demographic trends. According to UNICEF's *Generation 2030 Africa 2.0* [4], the continent's child population is projected to reach *1 billion by 2055*, comprising nearly 40% of the world's children. Meeting the health needs of this growing cohort will require ambitious scale‐up of essential services, including surgical care.

This study represents a pivotal step forward—not just in documenting need, but in highlighting opportunity. Pediatric surgical care is both *lifesaving and cost‐effective* [5], and should be integrated as a core component of child health programs and universal health coverage frameworks. The next phase must involve building congenital anomaly registries, investing in pediatric anesthesia, strengthening referral and emergency response systems, and expanding infrastructure beyond select urban centers.

As Hsu et al. rightly emphasize, this is not simply a report—it is a *call to action*. Policy‐makers, donors, and global health organizations must now treat pediatric surgery not as a luxury, but as a fundamental right of every child.

*Pediatric surgery is not optional. It is essential.*

## Author Contributions

**Zipporah Gathuya:** writing – original draft, writing – review and editing. **Elizabeth T. Drum:** conceptualization, writing – original draft, writing – review and editing.

## Conflicts of Interest

Elizabeth T. Drum: Member American Society of Anesthesiologists Charitable Foundation Board and Chair, Global Health Committee; World Federation of Societies of Anaesthesiologists Member of Council; American Academy of Pediatrics Section of Anesthesiology and Pain Medicine Executive Committee member and Vice‐Chair Elect Zipporah Gathuya: Global Initiative for Children's Surgery Board member, Smile Train Board Member.

## Data Availability

The authors have nothing to report.
